# Value of Perioperative Chest X-ray for the Prediction of Sternal Wound Complications after Cardiac Surgery in High-Risk Patients: A “Work in Progress” Analysis

**DOI:** 10.3390/jcm10020207

**Published:** 2021-01-08

**Authors:** Andrea Ardigò, Alessandra Francica, Gian Franco Veraldi, Ilaria Tropea, Filippo Tonelli, Cecilia Rossetti, Francesco Onorati, Giuseppe Faggian

**Affiliations:** 1 Division of Cardiac Surgery, University of Verona Medical School, 37126 Verona, Italy; andrea.ardigo@studenti.univr.it (A.A.); alessandrafrancica@yahoo.it (A.F.); ilariatropea1991@gmail.com (I.T.); filipp0tonelli92@gmail.com (F.T.); rossetticeci93@gmail.com (C.R.); giuseppe.faggian@univr.it (G.F.); 2 Vascular Surgery Unit, University Hospital in Verona, 37126 Verona, Italy; gianfranco.veraldi@aovr.veneto.it

**Keywords:** sternal dehiscence, surgical site infection, cardiac surgery, sternal synthesis

## Abstract

Background. Sternal wound complications are serious events that occur after cardiac surgery. Few studies have investigated the predictive value of chest X-ray radiological measurements for sternal complications. Methods. Several perioperative radiological measurements at chest X-ray and clinical characteristics were computed in 849 patients deemed at high risk for sternal dehiscence (SD) or More than Grade 1 Surgical Site Infection (MG1-SSI). Multivariable analysis identified independent predictors, whilst receiver operating characteristics (ROC) curve analyses highlighted cut-off values of radiological measurements for the prediction of both complications. Results. SD occurred in 8.8% of the patients, MG1-SSI in 6.8%. Chronic obstructive pulmonary disease (COPD) was the only independent predictor for SD (Odds Ratio, O.R. 12.1; *p* < 0.001); proximal sternal height (PSH) was the only independent protective factor (O.R. 0.58; *p* < 0.001), with a cut-off value of 11.7 mm (sensitivity 70.5%, specificity 71.0%; ROC area under the curve (AUC) = 0.768, *p* < 0.001). Diabetes mellitus (O.R. 3.5; *p* < 0.001) and COPD (O.R. 21.3; *p* < 0.001) were independent predictors for MG1-SSI; indexed proximal sternal height (iPSH) was as a protective factor (O.R. 0.26; *p* < 0.001) with a cut-off of 5.97 mm (sensitivity 70.2%, specificity 69.0%; ROC AUC = 0.739, *p* < 0.001). No other radiological measurements were independently correlated with SD or MG1-SS (*p* = N.S.). Conclusion. PSH and iPSH at preoperative chest X-ray may act as indicators of high risk for sternal wound complications, allowing for early preventative measures.

## 1. Introduction

Sternal dehiscence (SD) is a rare but potentially ill and painful complication that can occur after cardiac surgery, whose incidence is estimated to be between 0.2% to 3% [1]. Despite its low incidence, SD has a relevant effect on healthcare outcomes. In-hospital mortality has been reported between 10% and 14.2% [2,3,4,5] and the mean length-of-stay up to 33 days [4,5]. One- and five-year survival has been estimated at 72.4% ± 4.4% and 55.8% ± 5.6%, respectively [4]. Excess treatment costs are also reported [4,5]. To date, several comorbidities have been identified as clear independent predictors of SD [6], and different surgical techniques for sternal closure have been suggested across the decades to prevent it [7]. In addition, some postoperative radiological stigmas (e.g., sternal gap >3 mm or sternal wire migration/fracture on chest X-ray or substernal fluid and air in computed tomography) were demonstrated to confirm the occurrence of postoperative SD [8,9]. However, little attention to date has been paid to the existence of perioperative radiological data that can predict the risk of postoperative SD.

The chest X-ray has been a routine preoperative and postoperative examination after cardiac surgery since the dawn of this surgical specialty. The occurrence of SD has been early recognized as a risky complication, and different radiological examinations have been suggested to anticipate the clinical manifestation of SD [8,9]. Despite that, little attention has been paid to the potential predictive role of a routine chest X-ray. As an example, osteoporosis is considered a risk factor [6,10], and a biomechanical test with lateral sternal traction is reputed as the gold-standard to assess the tightness of different techniques of sternal closure [6,11]. Nevertheless, only one study has analyzed preoperative sternal thickness in terms of postoperative SD, with conflicting evidence [10]. Apart from sternal thickness, chronic obstructive pulmonary disease (COPD) and several functional respiratory parameters have been suggested as important risk factors for SD, but their radiological correlates have not been investigated [12,13,14,15,16].

According to the data mentioned above and to analyze the potential predictive role of chest X-ray on postoperative SD, we decided to collect data related to preoperative sternal thickness and perioperative height of different intercostal spaces (as indirect sign of hyperinflation and/or advanced COPD [15,16]) and correlate them with the postoperative development of SD. 

## 2. Materials and Methods

### 2.1. Patients

The retrospective study included consecutive patients considered at high risk for wound complications, who underwent surgery with sternotomy access from January 2018 to September 2020. Preoperative and postoperative characteristics were collected and used as criteria to identify high-risk patients for wound complications. Each patient met at least three high-risk factors for wound complications and surgical site infection according to published literature data [1,2,3,4,5].

### 2.2. Institutional Protocol

All patients underwent shaving the day before surgery and had a shower with chlorhexidine (CITROclorex 2% MD; Ecolab S.r.l, Vimercate, MB, Italy) on the morning of the planned operation, according to the preoperative preparation policy of the Division of Cardiac Surgery Verona. The institutional protocol provides antibiotic prophylaxis with cefazolin (Cefazolina Teva; Teva Pharmaceutical Industries, Milano, MI, Italy) 2 g 15–45 min before skin incision (3 g if weight >120 kg), repeated every 3 h during the operation and every 8 h until 24 h postoperation. In the case of methicillin-resistant *Staphylococcus aureus* colonization, vancomycin 1 g (Zengac; Fisiopharma S.r.l, Palomonte, SA, Italy) is indicated.

The department protocol suggests the use of osteosynthesis with single interrupted steel wires (Monofilament stainless steel—Covidien llc, Mansfield, MA, USA) for patients considered at low risk for wound dehiscence, while the Robicsek technique is indicated in patients at high risk for wound complication. Therefore, all patients underwent primary sternal closure with the Robicsek technique [11] at the end of the index procedure, according to institutional guidelines.

All patients underwent preoperative chest X-ray (posteroanterior (PA) and left lateral (LL) projections) the morning before planned cardiac surgery, as well as postoperative chest X-ray at ICU arrival and on postoperative day (POD) one (PA projection). All patients had already been extubated on POD one.

### 2.3. Study Design

**Figure 1:**
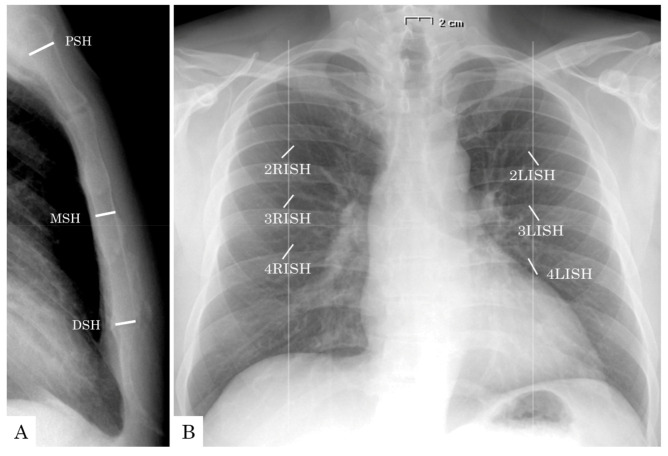
Perioperative measurements at chest X-ray. (**A**) PSH: proximal sternal height; DSH: distal sternal height; MSH: midsternal height. (**B**) 2LISH: 2nd left intercostal space height; 3LISH: 3rd left intercostal space height; 4LISH: 4th left intercostal space height; 2RISH: 2nd right intercostal space height; 3RISH: 3rd right intercostal space height; 4RISH: 4th right intercostal space height.

**Figure 2:**
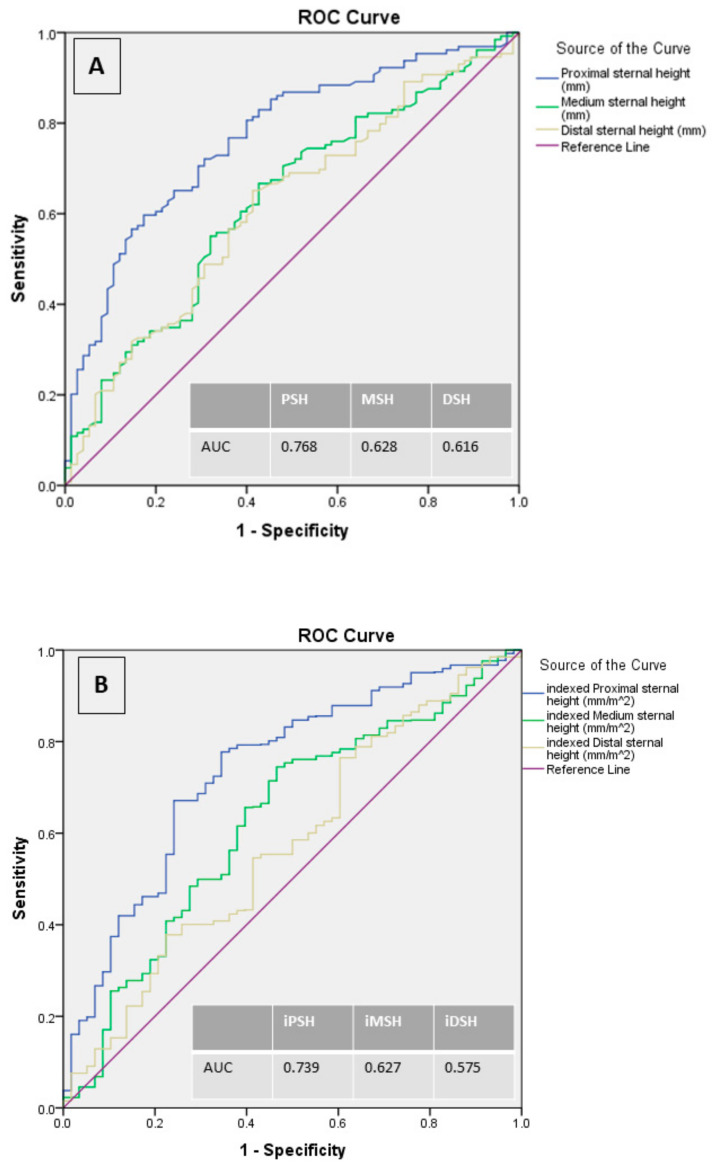
Receiver operating characteristics (ROC) curve models and the corresponding area under the curve (AUC) for sternal measurements in relation to sternal dehiscence (panel **A**) and indexed sternal measurements in relation to more than Grade 1 Surgical Site Infection (panel **B**).

For the purpose of the study, besides preoperative and perioperative baseline characteristics, several measurements were retrospectively computed from preoperative chest X-ray (PA and LL projections) and the 1st POD X-ray (AP projection). More specifically (1) maximal sternal height at manubrium (proximal sternal height, PSH), at mid-body (medium sternal height, MSH), and at the level of body–xiphoid process junction (distal sternal height, DSH) measured from preoperative X-ray in LL projection (Figure 1A); (2) the 2nd, 3rd and 4th left and right intercostal space height (pre-LISH and pre-RISH), measured at the level of the emiclavear line from preoperative X-ray in PA projection (Figure 1B); (3) the 2nd, 3rd and 4th left and right intercostal space height (post-LISH and post-RISH), measured at the level of the emiclavear line from 1st POD X-ray (Figure 1B). All of these measurements were collected as absolute values, as well as indexed measurements, after indexing for body surface area.

For the purpose of this study, the following primary endpoints were considered: (1) “SD”, defined as any mechanical instability perceivable during a physical examination of the patient within hospitalization or during the first 3 months after surgery, requiring surgical treatment or not; (2) “More than Grade 1 Surgical Site Infection (MG1-SSI)” according to 1999 Guidelines for the prevention of SSI [17]. Briefly, the guidelines differentiated between: (1) Grade 1 or “superficial site infection”, when infection occurs within 30 days after the operation and infection involves only skin or subcutaneous tissue of the incision; (2) Grade 2 or “deep incisional infection”, when infection occurs within 30 days after the operation if no implant is left in place, or within 1 year if an implant is in place, and the infection appears to be related to the operation and involves deep soft tissues (e.g., fascial and muscle layers) of the incision; (3) Grade 3 or “organ/space infection”, when infection occurs within 30 days after the operation if no implant is left in place, or within 1 year if an implant is in place, and the infection appears to be related to the operation and involves any part of the anatomy (e.g., organs or spaces), other than the incision, which was opened or manipulated during the operation [18]. 

The aim of this study was to recognize independent predictors of postoperative SD and MG1-SSI among all perioperative data, especially in the context of radiological measurements, in a population at high risk of wound complications.

The study protocol was approved by the Institutional Review Board (#prot.cscch 17/19; 18 November 2019). Informed consent was waived due to the retrospective nature of the study.

### 2.4. Inclusion and Exclusion Criteria

From January 2018 to September 2020, preoperative and perioperative data were retrospectively collected and used as criteria to identify patients at high risk for wound complications. In particular, patients presenting with at least three of the following perioperative parameters were considered at high risk for wound complications and surgical site infection: chronic obstructive pulmonary disease (COPD) ≥ 2 according to Global Initiative for Obstructive Lung Disease (GOLD) criteria [16], based on preoperative respiratory functional tests and/or specialist pneumologist consultation; diabetes mellitus with preoperative Hb1Ac > 53 mmol/mol [19]; obesity (BMI ≥ 30 kg/m^2^) [20]; chronic kidney disease; perioperative dialysis; bilateral internal mammary grafting (BIMA); re-exploration for bleeding; transfusions of > 4 units of red packed cells; postoperative tracheostomy in the first 48 h after surgery; prolonged ventilation > 96 h; and postoperative external cardiac massage [1,2,3,4,5]. All patients included in the study were extubated in POD one in order to avoid bias on hyperinflation due to mechanical ventilation.

To avoid potential confounders, emergent/urgent/salvage procedures, preoperative cardiopulmonary resuscitation, and preoperative Extra-Corporeal Membrane Oxygenation (ECMO) support were considered exclusion criteria.

### 2.5. Statistical Analysis

Statistical analysis was performed with SPSS for Windows (Version 15.0, SPSS Inc., Chicago, IL, USA). Continuous variables are presented as the mean and standard deviation, and categorical variables are presented as counts and percentages. The normal distribution of numerical variables was first assessed with the Shapiro–Wilk normality test. Normally distributed variables were compared with the unpaired *t*-test, whereas the Mann–Whitney U test was used for non-normally distributed variables. Categorical variables were analyzed using the chi-square test. Multivariable analysis was used to identify independent predictors of “SD”, as well as independent predictors of “MG1-SSI”. Stepwise logistic regression with backward selection was used for multivariable analyses. Only variables with a *p* < 0.10 at univariable analysis were included in the regression model to avoid overfitting. The Hosmer-Lemeshow test was used to assess the regression models fit. The area under the receiver operating characteristic curve (AUC) was used to represent the discriminatory ability of the regression models. The models are expressed in terms of adjusted odds ratio and 95% confidence interval (CI). A receiver operating characteristics (ROC) analysis was then calculated to determine optimal cut-off values for continuous variables acting as independent predictors of “SD” and of “MG1-SSI”. The area under the curve and its standard deviation (AUC SD), the sensitivity, and the specificity were calculated to analyze the diagnostic value of all these markers. A *p* value < 0.05 was considered significant for all statistical analyses.

## 3. Results

From January 2018 to September 2020, based on protocol definitions, a total of 75 (8.8%) SD and a total of 58 MG1-SSI (6.8%) were diagnosed on 849 consecutive patients considered at high risk for wound complications. The incidence of all-causes in-hospital mortality was 2.2% (*n* = 19), while only 0.2% (*n* = 2) was related to SD/MG1-SSI.

In the MG1-SSI group (*n*.tot = 58), 19% (*n* = 11) of patients underwent surgical debridement and mediastinal revision, while 81% (*n* = 47) required negative pressure wound therapy (NPWT therapy) with benefits.

In the SD group (*n*.tot = 75), 96% (*n* = 72) of patients were treated with NPWT therapy, while only 4% (*n* = 3) of patients was treated with iodoform gauze to promote secondary healing.

Baseline characteristics and intra- and post-operative data of the population are reported in Table 1. Principal outcomes are reported in Table 2.

Results of univariate analyses between patients developing SD and those with uncomplicated courses are reported in Table 3. 

When multivariable analysis was considered (AUC of the regression model = 0.76; 95% C.I. 0.68–0.84; Hosmer-Lemeshow test *p* = 0.28), COPD was the only risk-factor independently predicting SD among this high-risk cohort (O.R. 12.1; 95% C.I. 5.0–29.5; *p* < 0.001), whereas proximal sternal height was the only independent protective factor against SD (O.R. 0.58–95% C.I. 0.51–0.68; *p* < 0.001). Of note, receiver operating characteristics (ROC) analysis demonstrated PSH ≥ 11.7 mm as the best preoperative discriminator between SD and uncomplicated course with a sensitivity of 70.5% and a specificity of 71% (ROC AUC = 0.768, S.E. 0.027; *p* < 0.001–95% C.I. 0.72–0.82). Lower accuracy was reported for preoperative MSH and DSH (AUC of the ROC models = 0.628 and 0.616, respectively—Figure 2A).

Results of univariate analyses of patients developing MG1-SSI and those with uncomplicated courses are reported in Table 3 and Table S1 (view Supplementary Materials).

When MG1-SSI was considered at multivariable analysis (AUC of the regression model = 0.74; 95% C.I. 0.66–0.82; Hosmer-Lemeshow test *p* = 0.44), diabetes mellitus (O.R. 3.5; 95% C.I. 1.7–6.9; *p* < 0.001) and COPD (O.R. 21.3; 95% C.I. 6.9–65.9; *p* < 0.001) were identified as independent predictors of the outcome variable, whereas indexed proximal sternal height (iPSH) was the only protective factor against the event (O.R. 0.26; 95% C.I. 0.16–0.42; *p* < 0.001). Of note, ROC analysis demonstrated iPSH ≥ 5.97 mm as the best preoperative discriminator between MG1-SSI and uncomplicated course with a sensitivity of 70.2% and a specificity of 69.0% (ROC AUC = 0.739, S.E. 0.034; *p* < 0.001–95% C.I. 0.67–0.80). Lower accuracy was reported for preoperative iMSH and iDSH (AUC of the ROC models = 0.627 and 0.575, respectively—Figure 2B).

Finally, none of the other preoperative or postoperative radiological measurements were found to be independently correlated with SD or MG1-SSI at multivariable analyses (*p* = N.S. for all).

## 4. Discussion

The main finding of this study relates to the unequivocal demonstration that a simple preoperative radiological measurement of proximal sternal height can predict sternal complications after cardiac surgery in patients at high risk for wound complications. Furthermore, COPD was confirmed as the only independent predictor for SD, and both COPD and diabetes mellitus were found to be independent predictors for MG1-SSI. The latter results are in agreement with the previous literature [1,2,6,13,21]. On the other hand, the results concerning preoperative radiological measurements are interesting. More specifically, PSH and iPSH predicted SD and MG1-SSI, respectively, in our experience. Sternal tightness is universally considered a crucial factor for sternal wound healing. Previously identified risk-factors for SD, such as prior sternotomy [17], osteoporosis [22], asymmetric sternotomy [10], bilateral mammary artery grafting [1], obesity [6], discriminant use of electrocautery on the bone [23], early postoperative cardiopulmonary resuscitation [17], and large bra cups [24], all represent factors mining the integrity and stability of sternal reconstruction. Interestingly enough, despite the crucial role of sternum properties on its correct healing, little attention has been paid to date to radiological sternal characteristics on wound healing outcome data. To the best of our knowledge, only one study demonstrated that sternum thickness indexed to the bodyweight correlates with sternal instability at univariate analysis [10]. However, this study reported only 12 events (i.e., patients with sternal instability) in a limited number of patients (n. 171 patients enrolled), did not report any multivariable model, and did not specify where the maximal sternal thickness was reported out of the three different points of measurement. Additionally, measurements were taken intraoperatively before sternal closure [10]. Otherwise, we report a preoperative radiological measurement able to predict the complication, possibly leading to further preventative measurements against it. Moreover, we identify the exact location of our radiological measurements, leading to high reproducibility of our experience, especially in light of the fact that chest X-rays represent a first-level perioperative examination worldwide. Finally, we provide a precise cut-off value with acceptable specificity and sensibility in predicting either SD and MG1-SSI. 

Indeed, our findings suggest that the most important part in sternal tightness can be focalized at the proximal sternum (manubrium) since ROC analysis demonstrated a cut-off value of 11.7 mm for PSH and 5.97 mm for iPSH as protective independent factors for SD and MG1-SSI, respectively. This strictly depends on sternal thickness. This finding confirms previous biomechanical studies demonstrating that rotational moments, generated by distracting forces, primarily act at the top of the manubrium [25]. Moreover, it has been demonstrated that small changes in sternal thickness result in large changes in circumferential stress, and areas with small sternal thickness are the first interested by a dehiscent process (e.g., the sternal body/xiphoid process) [26]. Therefore, we can also infer that patients with low proximal sternal height might chiefly benefit from “reinforced” techniques of sternal wound closure, such as rigid sternal plate fixation, weave techniques [7], peristernal or sternal band closure techniques [27], or parasternal cable systems [28]. It has been proven that all these techniques better distribute the distraction forces acting across the sternal reconstruction, either at rest or during cough and Valsalva maneuvers, compared to single interrupted steel wires. 

Our incidence of sternal wound complications might be considered higher than the reported 0.2–3.0% of the literature [1], being the incidence of SD 8.8% and that of MG1-SSI 6.8% in this experience. However, most previous reports analyzed a widely heterogeneous population (from low to high risk) [1,6,9,10,14,28], whereas our study only investigated patients at high risk of wound complication, given the existence of at least three different risk-factors in each patient enrolled in the study. As an example, a study investigating only the role of asymmetric sternotomy found SD to develop in 7% of patients [10]. Furthermore, previous experiences did not specify the employed surgical techniques for sternal closure [1,6,9,14,17,29], while other studies only compared standard techniques of sternal closure with differently reinforced techniques of sternal synthesis [10,11,27,30,31]. This leads to introducing a strong bias in the interpretation of the results. Otherwise, our study investigates the topic using always the same technique of reinforced sternal closure (i.e., the Robicsek technique), thus analyzing a homogenous population of patients in terms of the employed surgical technique for chest closure.

Furthermore, among preoperative comorbidities, the strongest independent predictor of sternal complications in our series was COPD for both SD and MG1-SSI. This finding is not new [1,7,8,9,10] and has been correlated with COPD-derived tissue hypoxia [32] and re-exacerbation of cough in the perioperative period, causing increased stresses along the sternal wires with wire breakage and sternal bone fracture [33]. Again, our data suggest that COPD patients might benefit the most from reinforcement techniques of sternal closure, especially if a low sternal height is demonstrated at preoperative X-ray.

As previously reported in the literature, postoperative SD has relevant clinical implications. Despite its low incidence [1], it significantly increases 30-day and 1-year mortality rates [2,3,4,5], reduces long-term survival [4], prolongs hospital length of stay [4,5], and increases treatment costs [4,5]. Our retrospective study selected a high-risk population for SD or MG1-SSI, including all patients with at least three risk factors for wound/sternal dehiscence. These are mainly COPD, diabetes, and obesity, which have become increasingly common in patients undergoing cardiac surgery in recent decades [1,2,4,6,13]. Therefore, we think it is important to have a simple and high reproducible instrument, such as measurement of proximal sternal height at preoperative chest X-ray, which can predict the risk of SD. In this way, being able to preoperatively recognize patients at high risk of dehiscence, preventive measures could be implemented before surgery (e.g., optimizing COPD and diabetes therapies, reducing or stopping smoke, optimizing diet, or implementing therapy for osteoporosis). Similarly, it may be possible to determine which patients would benefit from “reinforced” techniques of sternal closure [7,27,31] and require special attention in the postoperative condition. Accordingly, several postoperative strategies could be implemented in these high-risk patients (e.g., tight glycemic control, thorax vest support, targeted rehabilitation, etc.).

In conclusion, simply by using a preoperative chest X-ray measurement, we were able to identify patients at high risk of sternal wound complications, in whom preventative strategies could be implemented. This may enable us to demonstrate a reduced incidence of these complications in future studies.

### Study Limitation

This is a retrospective study. The fairly small size of the population is due to the restrictive inclusion criteria that identify only patients at high risk for wound/sternal dehiscence. For the same reason, all patients had sternal closure with the Robicsek technique, according to the Institutional Guidelines of the Division of Cardiac Surgery Verona. Another limitation is the absence of previous literature studies addressing a potential predictive role of LISH/RISH on sternal complications. However, given their strict correlation with advanced COPD, which is a well-known predictor of wound complications, we aimed to analyze, for the first time, the eventual predictive value of this simple radiological measurement.

Data regarding the long-term outcomes were not collected for this study, as well as microbiology data. It could be interesting to collect these at a later date for further studies.

## Figures and Tables

**Table 1:** Baseline characteristics and perioperative data of the population.

Variable	Value
Age	67.0 ± 11.5
Sex (Female)	142 (16.7%)
EuroSCORE-II	3.5 ± 2.2
LVEF (%)	47.3 ± 13.0
Diabetes Mellitus	185 (21.8%)
Hb1Ac > 53 mmol/mol	105 (12.4%)
COPD	25 (2.9%)
Obesity	213 (25.1%)
BMI (Kg/m^2^)	27.3 ± 4.3
Chronic Kidney Disease	261 (30.7%)
Preoperative dialysis	41 (4.8%)
eGFR (ml/min/m^2^)	79.5 ± 18.8
Peripheral arteriopathy	122 (14.4%)
Redo	3 (0.5%)
NYHA class	2.4 ± 0.6
BIMA	3 (0.5%)
SIMA	250 (29.5%)
CABG	253 (29.8%)
Valve surgery	548 (64.5%)
Other type of cardiac surgery *	48 (5.7%)
ACC time (mins)	81.5 ± 40.2
CPB time (mins)	110.0 ± 52.6
Transfusion > 4 Units RPC	178 (21.0%)
Re-exploration for bleeding	33 (3.9%)
Prolonged ventilation (>96 h)	72 (8.5%)
Postoperative tracheostomy	12 (1.4%)
Postoperative CPR	7 (0.8%)

ACC: aortic cross-clamp; BIMA: bilateral internal mammary grafting; CABG: coronary artery bypass grafting; COPD: chronic obstructive pulmonary disease; CPB: cardiopulmonary bypass; CPR: cardiopulmonary resuscitation; eGFR: estimated Glomerular Filtration Rate; LVEF: left ventricular ejection fraction; SIMA: single mammary artery grafting; RPC: red packed cells. * Including ascending aorta procedures and all combined procedures.

**Table 2:** Principal outcomes in SD and MG1-SSI groups.

Variable	SD (75 Patients)	No SD (774 Patients)	*p*	MG1-SSI (58 Patients)	No MG1-SSI (791 Patients)	*p*
NPWT	72 (96%)	0 (-)	<0.001	47 (81%)	25 (3.1%)	<0.001
Surgical debridement	11 (14%)	0 (-)	<0.001	11 (19%)	0 (-)	<0.001
LOS	12.2 ± 17.5	6.1 ± 3.4	<0.001	13.2 ± 18.1	8.3 ± 11.4	0.0027
Death	2 (2.6%)	17 (2.2%)	0.6822	2 (3.4%)	17 (2.2%)	0.3771

NPTW: negative pressure wound therapy; LOS: length-of-stay.

**Table 3:** Univariate analysis of clinical data and radiological measurements between patients developing SD and MG1-SSI or not.

Variable	SD (75 Patients)	No SD (774 Patients)	*p*	MG1-SSI (58 Patients)	No MG1-SSI (791 Patients)	*p*
Age	68.0 ± 11.9	66.9 ± 11.5	0.391	69.2 ± 9.9	66.8 ± 11.6	0.137
Sex (Female)	47 (62.7%)	660 (85.3%)	<0.001	22 (37.9%)	120 (15.2%)	<0.001
EuroSCORE-II	4.7 ± 3.6	3.4 ± 2.0	<0.001	4.0 ± 3.5	3.5 ± 2.1	0.088
LVEF (%)	47.1 ± 14.9	47.3 ± 12.8	0.907	46.7 ± 16.6	47.4 ± 12.7	0.707
Diabetes Mellitus	29 (38.7%)	156 (20.2%)	<0.001	23 (39.7%)	162 (20.5%)	0.001
Hb1Ac > 53 mmol/mol	11 (14.7%)	94 (12.1%)	0.526	11 (19.0%)	94 (11.9%)	0.114
COPD	13 (17.3%)	12 (1.6%)	<0.001	11 (19.0%)	14 (1.8%)	<0.001
Obesity	27 (36.0%)	186 (24.0%)	0.022	20 (34.5%)	193 (24.4%)	0.087
BMI (Kg/m^2^)	28.4 ± 4.3	27.2 ± 4.3	0.016	28.3 ± 4.3	27.2 ± 4.3	0.055
Chronic Kidney Disease	23 (30.7%)	238 (30.7%)	0.988	23 (39.7%)	238 (30.1%)	0.127
Preoperative dialysis	6 (8.0%)	35 (4.5%)	0.180	6 (10.3%)	35 (4.4%)	0.042
eGFR (ml/min/m^2^)	67.4 ± 25.7	80.7 ± 17.6	<0.001	65.2 ± 25.7	80.6 ± 17.8	<0.001
Peripheral arteriopathy	18 (24.0%)	104 (13.4%)	0.013	18 (31.0%)	104 (13.1%)	<0.001
Redo	3 (4.0%)	0 (-)	<0.001	3 (5.2%)	0 (-)	<.001
NYHA class	2.3 ± 0.7	2.5 ± 0.6	0.076	2.4 ± 0.7	2.4 ± 0.6	0.691
BIMA	3 (4.0%)	0 (-)	<0.001	3 (5.2%)	0 (-)	<0.001
SIMA	31 (41.3%)	219 (28.3%)	0.018	31 (53.4%)	219 (27.7%)	<0.001
CABG	34 (45.3%)	219 (28.3%)	0.002	34 (58.6%)	219 (27.7%)	<0.001
Valve surgery	38 (50.7%)	510 (65.9%)	0.008	21 (36.2%)	527 (66.6%)	<0.001
Other type of cardiac surgery *	3 (4.0%)	45 (5.8%)	0.516	3 (5.2%)	45 (5.7%)	0.869
ACC time (mins)	67.1 ± 33.8	82.9 ± 40.5	0.001	67.4 ± 36.1	82.6 ± 40.3	0.005
CPB time (mins)	101.9 ± 52.5	110.8 ± 52.6	0.163	104.6 ± 57.6	110.4 ± 52.2	0.416
Transfusion > 4 Units RPC	17 (22.7%)	161 (20.8%)	0.705	13 (22.4%)	165 (20.9%)	0.779
Re-exploration for bleeding	4 (5.3%)	29 (3.7%)	0.497	4 (6.9%)	29 (3.7%)	0.219
Prolonged ventilation (>96 h)	6 (8.0%)	66 (8.5%)	0.876	6 (10.3%)	66 (8.3%)	0.598
Postoperative tracheostomy	2 (2.7%)	10 (1.3%)	0.336	2 (3.4%)	10 (1.3%)	0.174
Postoperative CPR	2 (2.7%)	5 (0.6%)	0.065	2 (3.4%)	5 (0.6%)	0.022
PSH (mm)	10.8 ± 1.7	12.7 ± 1.9	<0.001	10.8 ± 1.7	12.6 ± 1.9	<0.001
MSH (mm)	9.5 ± 1.7	10.3 ± 1.9	<0.001	9.4 ± 1.8	10.3 ± 1.9	0.001
DSH (mm)	9.5 ± 1.8	10.3 ± 2.1	0.002	9.6 ± 1.9	10.3 ± 2.2	0.016
iPSH (mm/m^2^)	5.8 ± 0.9	6.7 ± 1.1	<0.001	5.7 ± 1.0	6.6 ± 1.1	<0.001
iMSH (mm/m^2^)	5.1 ± 1.0	5.4 ± 1.0	0.005	5.0 ± 1.0	5.4 ± 0.9	0.003
iDSH (mm/m^2^)	5.1 ± 1.1	5.4 ± 1.1	0.021	5.1 ± 1.1	5.4 ± 1.1	0.059

ACC: aortic cross-clamp; BIMA: bilateral internal mammary grafting; CABG: coronary artery bypass grafting; COPD: chronic obstructive pulmonary disease; CPB: cardiopulmonary bypass; CPR: cardiopulmonary resuscitation; iDSH: indexed distal sternal height; iMSH: indexed midsternal height; iPSH: indexed proximal sternal height; LVEF: left ventricular ejection fraction; DSH: distal sternal height; MSH: midsternal height; PSH: proximal sternal height; RPC: red packed cells; SD: sternal dehiscence; SIMA: single mammary artery grafting. * Including ascending aorta procedures and all combined procedures.

## Data Availability

Data is contained within the article or supplementary material.

## Supplementary Materials

The following are available online at https://www.mdpi.com/2077-0383/10/2/207/s1, Table S1: Univariate analysis of additional radiological measurements between patients developing or not SD and MG1-SSI.
