# Supplementary material for: Value of Perioperative Chest X-ray for the Prediction of Sternal Wound Complications after Cardiac Surgery in High-Risk Patients: A “Work in Progress” Analysis
Source: J Clin Med. 2021 Jan 8;10(2):207. doi: 10.3390/jcm10020207 (PMC7827951; doi:10.3390/jcm10020207)
Supplement: Supplementary file 1 [file jcm-10-00207-s001.pdf]

Journal of
Clinical Medicine

Table supplement. Univariate analysis of additional radiological measurements between patients developing or not SD and MG1-SSI.

Variable	SD (75 patients)	No SD (774 patients)	*p*	MG1-SSI (58 patients)	No MG1-SSI (791 patients)	*p*
pre-2LISH (mm)	19.8±3.9	18.5±4.5	0.026	20.0±5.3	18.6±4.5	0.020
pre-3LISH (mm)	18.9±3.9	18.7±3.7	0.572	18.8±3.9	18.7±3.7	0.852
pre-4LISH (mm)	19.7±3.8	18.4±4.2	0.008	19.5±3.8	18.5±4.1	0.069
pre-2RISH (mm)	20.8±5.1	19.8±5.3	0.110	20.6±4.9	19.9±5.3	0.325
pre-3RISH (mm)	21.1±4.4	19.7±4.6	0.015	20.8±4.3	19.8±4.6	0.110
pre-4RISH (mm)	20.1±4.9	18.9±3.8	0.010	20.1±5.2	18.9±3.8	0.031
post-2LISH (mm)	21.3±5.7	20.4±5.3	0.119	20.9±5.7	20.4±5.3	0.493
post-3LISH (mm)	19.8±4.7	18.9±4.5	0.076	19.7±4.7	18.9±4.5	0.219
post-4LISH (mm)	20.9±5.3	18.5±4.0	<0.001	20.8±5.3	18.6±4.0	<0.001
post-2RISH (mm)	22.2±5.5	21.5±5.9	0.337	22.3±5.5	21.5±5.9	0.354
post-3RISH (mm)	21.3±4.2	20.4±4.9	0.143	20.9±4.3	20.5±4.8	0.443
post-4RISH (mm)	21.3±5.7	18.7±4.1	<0.001	20.7±5.6	18.8±4.2	0.001
ipre-2LISH (mm/m^2)	10.7±3.2	9.8±2.6	0.007	10.7±3.4	9.8±2.6	0.009
ipre-3LISH (mm/m^2)	10.1±2.1	9.8±2.6	0.281	10.0±2.2	9.8±2.2	0.610
ipre-4LISH (mm/m^2)	10.5±2.0	9.7±2.3	0.002	10.4±2.1	9.7±2.3	0.037
ipre-2RISH (mm/m^2)	11.2±2.9	10.5±3.0	0.047	11.0±2.8	10.5±3.0	0.228
ipre-3RISH (mm/m^2)	11.3±2.5	10.4±2.6	0.003	11.1±2.6	10.4±2.6	0.048
ipre-4RISH (mm/m^2)	10.7±2.5	9.9±2.2	0.003	10.7±2.7	9.9±2.1	0.021
ipost-2LISH (mm/m^2)	11.4±3.3	10.7±3.0	0.049	11.2±3.4	10.7±3.0	0.319
ipost-3LISH (mm/m^2)	10.7±2.8	9.9±2.5	0.016	10.5±2.8	9.9±2.5	0.093
ipost-4LISH (mm/m^2)	11.2±2.9	9.8±2.2	<0.001	11.0±2.9	9.8±2.3	<0.001
ipost-2RISH (mm/m^2)	11.9±3.2	11.4±3.4	0.178	11.9±3.2	11.4±3.4	0.253
ipost-3RISH (mm/m^2)	11.5±2.7	10.7±2.6	0.020	11.2±2.8	10.8±2.6	0.159
ipost-4RISH (mm/m^2)	11.3±3.0	9.8±2.2	0.020	11.0±3.0	9.9±2.3	<.001

ipost-2LISH: indexed 2nd left intercostal space height; ipost-3LISH: indexed 3rd left intercostal space height; ipost-4LISH: indexed 4th left intercostal space height; ipost-2RISH: indexed 2nd right intercostal space height; ipost-3RISH: indexed 3rd right intercostal space height; ipost-4RISH: indexed 4th right intercostal space height; ipre-2LISH: indexed 2nd left intercostal space height; ipre-3LISH: indexed 3rd left intercostal space height; ipre-4LISH: indexed 4th left intercostal space height; ipre-2RISH: indexed 2nd right intercostal space height; ipre-3RISH: indexed 3rd right intercostal space height; ipre-4RISH: indexed 4th right intercostal space height; post-2LISH: 2nd left intercostal space height; post-3LISH: 3rd left intercostal space height; post-4LISH: 4th left intercostal space height; post-2RISH: 2nd right intercostal space height; post-3RISH: 3rd right intercostal space height; post-4RISH: 4th right intercostal space height; pre-2LISH: 2nd left intercostal space height; pre-3LISH: 3rd left intercostal space height; pre-4LISH: 4th left intercostal space height; pre-2RISH: 2nd right intercostal space height; pre-3RISH: 3rd right intercostal space height; pre-4RISH: 4th right intercostal space height.
